# Characterization of Osteoarthritis in a Medial Meniscectomy-Induced Animal Model Using Contrast-Enhanced X-ray Microtomography

**DOI:** 10.3390/biomedicines8030056

**Published:** 2020-03-10

**Authors:** Takehito Sugasawa, Tomoaki Kuji, Kai Aoki, Koki Yanazawa, Akiko Takenouchi, Makoto Watanabe, Yoshiya Tome, Yoshinori Takeuchi, Yuichi Aita, Naoya Yahagi, Yasuhiro Shishikura, Seiko Ono, Yasuko Yoshida, Yasushi Kawakami, Kazuhiro Takekoshi

**Affiliations:** 1 Laboratory of Laboratory/Sports Medicine, Division of Clinical Medicine, Faculty of Medicine, University of Tsukuba, 1-1-1 Tennodai, Tsukuba, Ibaraki 305-8577, Japan; take0716@krf.biglobe.ne.jp (T.S.); jimbeamilkybonbon330@gmail.com (S.O.); y-kawa@md.tsukuba.ac.jp (Y.K.); 2 Doctoral Program in Sports Medicine, Graduate School of Comprehensive Human Sciences, University of Tsukuba, 1-1-1 Tennodai, Tsukuba, Ibaraki 305-8577, Japan; s1930394@s.tsukuba.ac.jp (T.K.); fineday0126@gmail.com (K.A.); 3 Master’s Program in Medical Sciences, Graduate School of Comprehensive Human Sciences, University of Tsukuba, T1-1-1 Tennodai, Tsukuba, Ibaraki 305-8577, Japan; s1921312@s.tsukuba.ac.jp (K.Y.); s1921290@s.tsukuba.ac.jp (Y.S.); 4 Bonding and Manufacturing Field, Research Center for Structural Materials, National Institute for Materials Science, 1-2-1 Sengen, Tsukuba, Ibaraki 305-0047, Japan; TAKENOUCHI.Akiko@nims.go.jp (A.T.); WATANABE.Makoto@nims.go.jp (M.W.); 5 Research Center for Advanced Science and Technology, The University of Tokyo, 4-6-1 Komaba, Meguro-ku, Tokyo 153-8904, Japan; 6 Department of Medical Technology, Faculty of Health Sciences, Tsukuba International University, 6-20-1 Manabe, Tsuchiura, Ibaraki 300-0051, Japan; tome@tius.ac.jp (Y.T.); y-yoshida@tius.ac.jp (Y.Y.); 7 Nutrigenomics Research Group, Faculty of Medicine, University of Tsukuba, 1-1-1 Tennodai, Tsukuba, Ibaraki 305-8577, Japan; yoshinori-takeuchi@umin.ac.jp (Y.T.); metabmetabmetab@gmail.com (Y.A.); nyahagi-tky@umin.ac.jp (N.Y.)

**Keywords:** osteoarthritis, synovitis, articular cartilage, microfocus X-ray CT, 3D analysis

## Abstract

The aim of this study was to clarify degradation characteristics in each tissue of the knee complex of a medial meniscectomy (MMx)-induced knee osteoarthritis (KOA) animal model using classical methods and an alternative comprehensive evaluation method called contrast-enhanced X-ray micro-computed tomography (CEX-μCT), which was developed in the study. Surgical MMx was performed in the right knee joints of five male Wistar rats to induce KOA. At four weeks post-surgery, the synovitis was evaluated using quantitative polymerase chain reaction (qPCR). Degradations of the articular cartilage of the tibial plateau were evaluated using classical methods and CEX-μCT. Evaluation of the synovitis demonstrated significantly increased expression levels of inflammation-associated marker genes in MMx-treated knees compared with those in sham-treated knees. Evaluation of the articular cartilage using classical methods showed that MMx fully induced degradation of the cartilage. Evaluation using CEX-μCT showed that local areas of the medial cartilage of the tibial plateau were significantly reduced in MMx-treated knees compared with those in sham-treated knees. On the other hand, total cartilage volumes were significantly increased in MMx-treated knees. On the basis of the findings of this study, the method could be relevant to study new treatments in KOA research.

## 1. Introduction

The prevalence of osteoarthritis (OA) in humans has been rapidly increasing owing to an aging population and a rise in obesity rates [1]. OA is characterized by chronic pain, dysfunction of joint, and tissue degradation of joint cartilage and subchondral bone with synovitis [2]. Knee OA (KOA) causes high morbidity and is recognized as the most common type with a prevalence of 80% among all OA [1]. Advanced KOA causes severe pain and significant limitations in daily life [3]. For severe KOA, if the symptoms are not relieved by exercise and intra-articular injection of hyaluronic acid, knee replacement using an artificial knee joint is often performed [3].

Studies on the development of drugs to treat KOA are often performed using animal models. Evaluation of joint cartilage is considered critical as the primary tissue degradation in KOA animal models that mimic human KOA occurs in this tissue; however, many studies have been limited. For example, in some KOA animal studies, only local assessment of the cartilage has been done using tissue sections and staining methods to assess cartilage degeneration quantitatively [4,5,6,7]. In addition, microscopic evaluation using India ink staining to confirm cartilage degradation in KOA animal models has demonstrated localized positive and non-positive areas [8,9,10]. It is also difficult to excise a limited range of denaturation sites, such as India ink positive areas, during tissue sectioning for histological examination with precise targeting without a deviation of several μm. Furthermore, if tissue slice preparation fails, it cannot be redone. One way to solve this problem is to make dozens of tissue sections by microtome per sample with no fails, integrate them, and analyze them. However, this method takes a lot of time and remarkable technique and is not practical if dozens of samples are analyzed.

Furthermore, when evaluating multiple individuals, it is almost impossible to exactly excise the same part for all individuals, which results in poor reproducibility. Therefore, it is unclear whether lesion sites are accurately captured and evaluated using tissue staining methods.

It is necessary to perform a comprehensive evaluation of the KOA model, including its reproducibility, in order to understand the pathophysiology of cartilage degradation in detail. In addition, evaluation of subchondral bone and synovium is also important as these tissues are degraded in human KOA and KOA animal models [2]. Xie et al. [11] and Willett et al. [12] have previously described the method of equilibrium partitioning of an ionic contrast agent via microcomputed tomography (EPIC-μCT), which can quantify total and local volumes and determine the quality of articular cartilage in a small animal model with high precision and accuracy. However, the ionic compound ioxaglic acid (Hexabrix; Mallincrodt), which is the contrast agent used in this method, is difficult to obtain in Japan.

Therefore, in the current study, we developed a method using μCT and the nonionic alternative contrast agent iopamidol (Oypalomin; Fuji Pharma) to evaluate articular cartilage comprehensively. This method was named contrast-enhanced X-ray micro-computed tomography (CEX-μCT). The ancillary aim of the current study was to establish a foundation for CEX-μCT as a method to comprehensively evaluate articular cartilage. The primary aim was to apply CEX-μCT and classical methods to clarify the degradation characteristics of articular cartilage, subchondral bone, and synovium in the knee complex of a medial meniscectomy (MMx)-induced KOA animal model of post-traumatic OA.

## 2. Materials and Methods

### 2.1. Animal Experiments

Three male Wister rats (Japan SLC) at 10 weeks of age (242–283 g) were used to establish the foundation of the CEX-μCT methodology. The rats were euthanized by excess inhalation of isoflurane and the right and left knee complexes were then harvested for use as normal samples. The tissue specimens were immersed in 10% neutral buffered formalin solution (Fujifilm, Wako, Osaka, Japan) for 48 h at room temperature with gentle shaking. After 48 h of fixations, the samples (*n* = 6 knees) were subjected to analysis.

**Figure 1:**
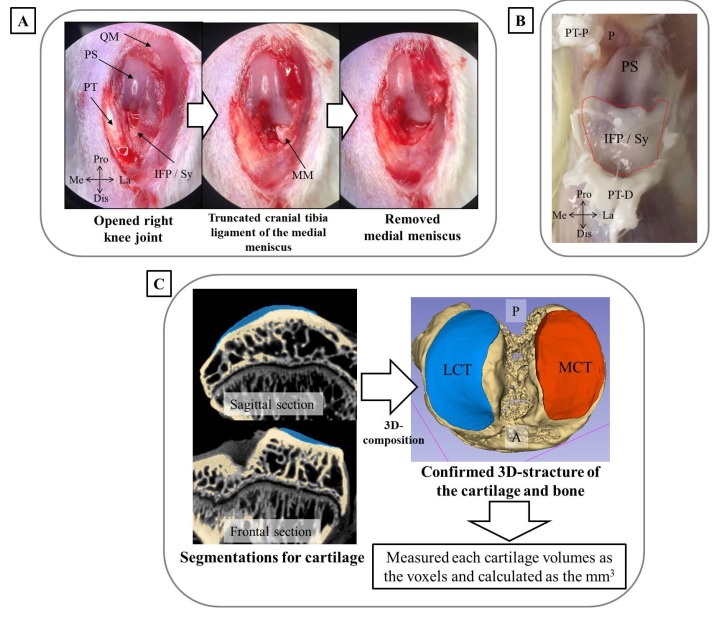
Experimental methodology used in the current study. (**A**) Surgery performed to establish medial meniscectomy (MMx)-induced knee osteoarthritis (KOA) in the right knee. The left figure shows the skin incision and internal knee joint with a laterally dislocated patella. The middle figure shows a destabilized meniscus generated by truncating the cranial tibia ligament of the medial meniscus. The right figure shows the removed meniscus of the part adhering to the medial collateral ligament. (**B**) The methodology used to harvest IFP and synovium. After removal of the patella tendon, the IFP (indicated by the red dotted line) was harvested using a surgical knife. (**C**) The methods for segmentation of the cartilage and subchondral bone and calculation of cartilage volume using contrast-enhanced X-ray micro-computed tomography (CEX-μCT) images and 3D slicer software. Abbreviations: QM, quadriceps muscle; PS, patellar surface; PT, patellar tendon; IFP, infrapatellar fat pad; Sy, synovium; Pro, proximal; Dis, distal; La, lateral; Me, medial; MM, medial meniscus; P, patella; PT-P, patellar tendon-proximal; PT-D, patellar tendon-distal; LCT, lateral articular cartilage of tibia plateau; MCT, medial articular cartilage of tibia plateau; A, anterior; P, posterior.

Another five male Wister rats at 5 months of age (371–410 g) underwent MMx surgery to establish the KOA model. The surgery was performed according to the method reported by Yanagisawa et al. [13]. Briefly, while anesthetized by isoflurane inhalation, a skin incision of approximately 2 cm was made in the right knee, the patella and patellar tendon were then laterally dislocated, and a joint capsule incision was made. The meniscus was destabilized by truncating the cranial tibia ligament, and the medial meniscus was removed by cutting the part adhering to the medial collateral ligament (Figure 1A). Finally, the fascia and the skin were stitched. For the left knee, which underwent sham surgery and was used as a control, only the incision was made. At four weeks post-surgery, the rats were euthanized by excess inhalation of isoflurane. After removal of the patella tendon, the synovium, including the infrapatellar fat pad (IFP), was harvested for evaluation of inflammatory marker genes and cartilage degradation (Figure 1B). The synovium was quickly placed in liquid nitrogen and stored at −80 °C until further analysis. The knee complex was also harvested and immersed to 10% neutral buffered formalin solution and incubated for 48 h at room temperature with gentle shaking. After 48 h of fixation, the knee complex underwent subsequent analysis. These animal experiments were approved by the Animal Care Committee, University of Tsukuba (approval numbers: 19-164, 1 June 2019).

### 2.2. Establishing the Foundation of the CEX-μCT Method

After fixation of the normal knee complexes, the knee joints were separated, and soft tissues were removed. The tibias, including the articular cartilage, was then immersed in Oypalomin (0%, 10%, 20%, 30%, 40%, and 50% each, total of six conditions) diluted with phosphate-buffered saline (PBS) and incubated overnight at room temperature. The following day, after thoroughly wiping the remaining liquid off of the tibias, the samples were placed in 15 mL centrifuge tubes and X-ray μCT scans were performed using an SMX-160CTS micro-focus X-ray system (Shimazu Corporation). The following apparatus parameter settings were used:

1. Source-to-image distance (SID): 200 mm
2. Source-to-object distance (SOD): 40 mm
3. Tube voltage: 47 kV
4. Number of views: 1200
5. Scaling coefficient: 1200
6. Image sharp: 512
7. Widthwise pixel spacing (mm/pixel): 0.021181
8. Lengthwise pixel spacing (mm/pixel): 0.021181
9. Saved image type: TIF 16-bit

The frontal section of articular cartilage and subchondral bone of tibias were analyzed using the volume viewer plugin of the ImageJ Fiji ver. 1.52p software [14]. The brightness of refracted X-ray for both the articular cartilage and subchondral bone within the region of interest (ROI) were also measured using the same software to determine the optimal concentration of Oypalomin to differentiate articular cartilage and subchondral bone of the tibia. The ROIs were manually determined at medial tibial plateaus containing articular cartilage and subchondral bone.

### 2.3. Evaluation of Subchondral Bone in MMx-Induced KOA Using X-ray μCT

After fixation of the MMx-induced KOA model knee complexes, they were subjected to X-ray μCT analysis to evaluate the degradation of subchondral bone. The samples were placed in 50 mL centrifuge tubes, and X-ray μCT analyses were performed using the same methods and parameters listed above, except the SOD was changed to 100 mm. The subchondral bone of the knee complex was imaged utilizing the volume viewer plugin of the ImageJ Fiji ver.1.52p software using the X-ray μCT image, the projection image, 3D images, and section image views [14]. The subchondral bone changes caused by MMx-induced KOA were morphologically analyzed.

### 2.4. CEX-μCT Analysis of MMx-Induced KOA

After performing X-ray μCT analysis of the subchondral bones from MMx-treated mice, the knee complexes were separated into the tibia fully removed soft tissues. On the basis of the results of Section 2.2, the optimal concentrations of Oypalomin was 30%. Therefore, the tibias from MMx-induced KOA mice were immersed overnight in 30% Oypalomin. The following day, after thoroughly wiping the remaining liquid off the tibias, the samples were placed in 15 mL centrifuge tubes, and X-ray μCT scans were performed as described in Section 2.2. The subchondral bone of the knee complex was imaged utilizing the volume viewer plugin of the ImageJ Fiji ver.1.52p software using the X-ray μCT image, the projection image, 3D images, and section image views [14]. Using the 3D images, we confirmed whether to reflect the form under the stereomicroscope.

Furthermore, the frontal section images were generated from the 3D images using the same plugin of the same software. The articular cartilage pixel area of the medial tibial plateau was measured and confirmed to correlate with the area of articular cartilage obtained by tissue staining and microscopic observation.

Segmentation analysis was also performed after the reconstruction of the 3D images using 3D slicer software ver. 4.10.1. with the Segment Editor plugin to differentiate the cartilage and subchondral bone. The total voxels of the articular cartilage were then measured using the Segment Statistics plugin in the same software. The total voxels were converted to a volume (mm^3^) to comprehensively evaluate the articular cartilage, which was could be used to quantify total cartilage volumes (Figure 1C).

### 2.5. Macroscopic Imaging of Articular Cartilage in MMx-Induced KOA

After performing the CEX-μCT analysis, macroscopic images were obtained to evaluate articular cartilage degradation with or without India ink stain. The osteophyte and gross finding score and India ink-positive area were measured according to the method of Yanagisawa et al. [13].

### 2.6. Histological Analyses

After macroscopic imaging, the tibias were immersed in Kalkitox decalcification solution (Fujifilm, Wako, Osaka, Japan) for 48 h at 4 °C. The samples were then embedded in paraffin, followed by neutralization with 5% sodium sulfate solution. Frontal sections (5 μm) of each sample were prepared that included a region of severe degradation. Safranin-O and Fast Green staining were then performed. Photomicrographs of the stained tissues were obtained on a BZ-X710 fluorescence microscope (Keyence, Osaka, Japan) and the Safranin-*O*-positive areas (pixels) were calculated using the ImageJ Fiji ver.1.52p software [14]. The Osteoarthritis Research Society International (OARSI) guideline scores [15] for the refraction of articular cartilage degradation were calculated in a double blind manner using the photomicrographs separated one-third on the medial side and two-thirds on the lateral side. The correlation between the articular cartilage areas (pixels) of the medial tibial plateau obtained by tissue staining and CEX-μCT method was confirmed to evaluate the accuracy of the CEX-μCT method.

### 2.7. Evaluation of Synovitis by Quantitative Real-Time Polymerase Chain Reaction (qPCR)

Synovitis-associated gene expression measured using qPCR. The synovium was homogenized in Sepasol-RNA I Super G (Nacalai Tesque, Kyoto, Japan), and total RNA was extracted according to the manufacturer’s protocol. Then, 500 ng of total RNA was subjected to reverse transcription using PrimeScrip RT Master Mix (Takara Bio, Shiga, Japan) according to the manufacturer’s protocol to generate complementary DNA (cDNA). The cDNA was serially diluted 10-fold with sterilized distilled water and qPCR was performed to detect the expression of synovitis-associated maker genes. The qPCR reaction mixture (10 µL) was prepared using a KAPA SYBR Fast qPCR Kit (Nippon Genetics, Tokyo, Japan) according to the manufacturer’s protocol. Specific primers were used at final concentrations of 100 nM. Amplification was performed using a QuantStudio 5 Real-Time PCR Systems thermal cycler (Thermo Fisher Scientific, Waltham, MA, USA). The cycling profile included one cycle of 95 °C for 5 min, followed by 40 cycles of 95 °C for 3 s and 60 °C for 30 s, with a final stage of melting curve analysis. Hypoxanthine phosphoribosyltransferase (HPRT) RNA expression was also measured and used as the internal sample control. The value of each gene expression was calculated using the 2^ΔΔ*C*t^ method and normalized to HPRT levels. The primer sequences and targeted gene names are shown in Supplemental Table S1.

### 2.8. Statistical Analyses

Data are presented as means ± standard deviation (SD). The paired *t*-test for the Sham (left) ersus MMx (right) knee was performed for comparison between two groups using Excel 2010 software (Microsoft). For the analysis of OARSI scores for four groups, the Bonferroni correction was performed as multiple comparisons. Pearson’s correlation coefficient on the area of articular cartilage between stained tissue and CEX-μCT analysis was also calculated using the same software. *p* < 0.05 was considered statistically significant.

## 3. Results

### 3.1. Optimal Oypalomin Concentration for CEX-μCT Was 30%

**Figure 2:**
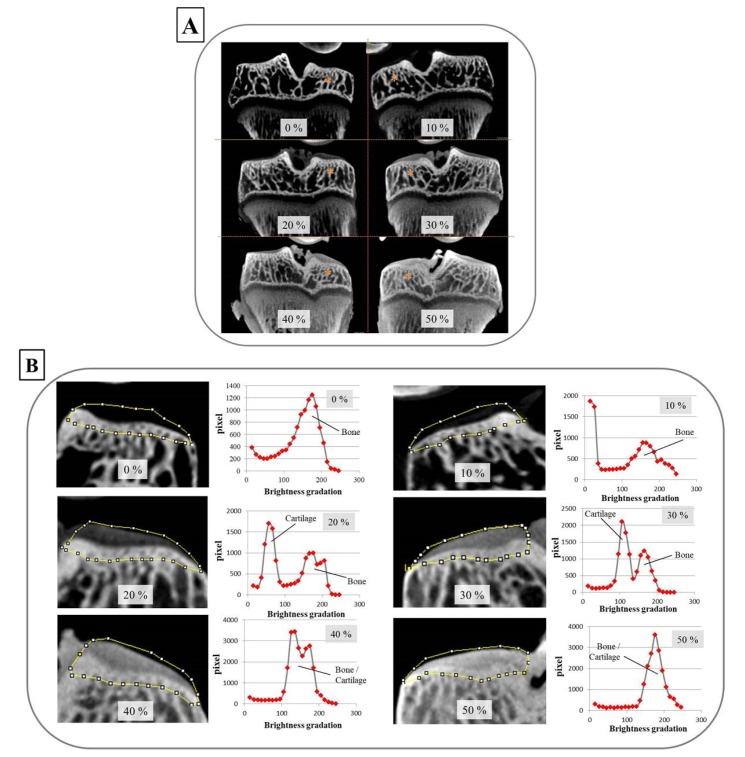
The optimal concentration of Oypalomin for CEX-μCT was 30%. (**A**) The overall image of tibia frontal sections with the indicated concentrations (%) of Oypalomin. Staining of the articular cartilage of the tibial plateau was dependent on the Oypalomin concentrations. The asterisks indicate the medial tibial condyle. (**B**) Brightness measurements reflected X-ray absorptions of the articular cartilage and subchondral bone. The regions of interest (ROIs) surrounded by the yellow line were determined manually. Brightness and pixels within the ROI are plotted as histograms. The optimal concentration of Oypalomin to differentiate cartilage and bone was 30%.

In establishing the foundation for the CEX-μCT method, articular cartilage from the tibia plateau was stained using various concentrations of Oypalomin. Neither 0% or 10% Oypalomin allowed for the depiction of articular cartilage from the medial tibial plateau. On the other hand, both 20% and 50% Oypalomin could allow for visualization of the articular cartilage and provided adequate contrast between the bone and cartilage (Figure 2A). Analysis of brightness refracted to determine the X-ray absorption differences between bone and cartilage demonstrated that 30% Oypalomin was the optimal concentration to differentiate cartilage and bone (Figure 2B).

### 3.2. Subchondral Bone Degradation Was Not Discovered by MMx-Induced KOA According to the X-ray μCT Analyses

**Figure 3:**
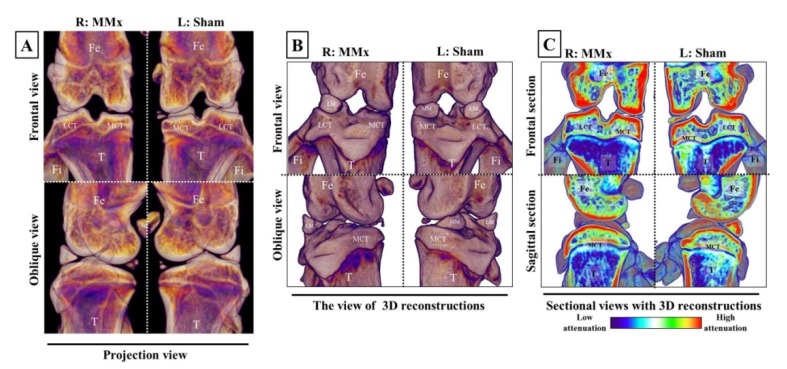
Images of subchondral bones in the knee complex on X-ray μCT analyses. The pictures show representative examples of projection (**A**), 3D (**B**), and sectional (**C**) views on X-ray μCT analyses. No changes in the subchondral bones of MMx-treated knees compared with that of sham-treated knees were noted. Abbreviations; Fe, femur; LCT, lateral condyle of tibia; MCT, medial condyle of tibia; T, tibia; Fi, fibula; LM, lateral meniscus; MM, medial meniscus.

In the evaluations of subchondral bone using X-ray μCT, the bone degradation was not morphologically discovered upon MMx-treated knees on the projection, 3D, or sectional views. The only notable observation was the disappearance of the meniscus of the right knee following MMx treatment (Figure 3A–C).

### 3.3. MMx-Treated Knees Showed Articular Cartilage Degradation on Macroscopic Imaging

**Figure 4:**
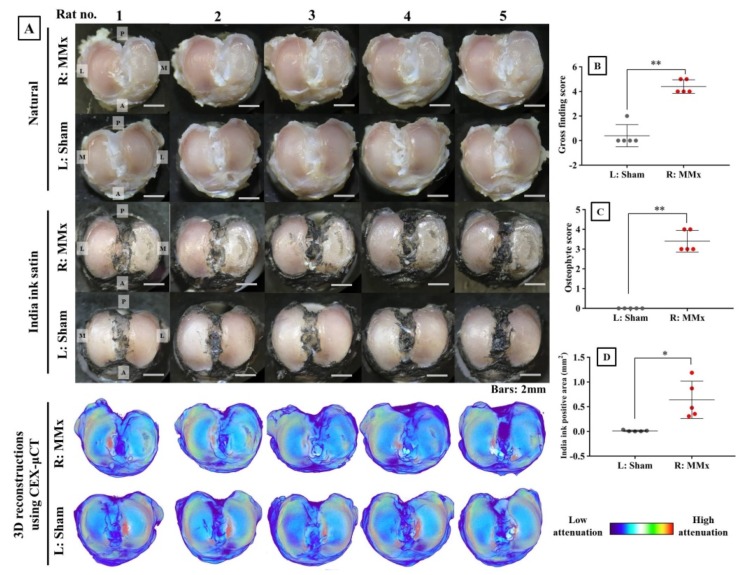
Analyses of macroscopic imaging and 3D reconstruction images using the CEX-μCT method. (**A**) Macroscopic images with or without India ink staining and 3D reconstruction images using the CEX-μCT method of all the samples. (**B**) Gross finding score for the articular cartilage stained by India ink. (**C**) Osteophyte score for the non-stained articular cartilage. (**D**) India ink-positive area for the articular cartilage stained by India ink. Abbreviations: P, posterior; A, anterior; M, medial; L, lateral. * *p* < 0.05, ** *p* < 0.01.

Analyses using macroscopic imaging with or without India ink stain fully confirmed the degradation of articular cartilage (Figure 4A). The gross finding scores, osteophyte scores, and India ink-positive areas were all significantly increased in MMx-treated knees compared with those in sham-treated knees (Figure 4B–D). In the 3D reconstruction of the articular cartilage using the CEX-μCT method, the cartilage degeneration corresponding to macroscopic images was morphologically faithfully reproduced (Figure 4A).

### 3.4. MMx-Treated Knees Showed Articular Cartilage Degradation Using Either Tissue Staining or CEX-μCT Analyses

**Figure 5:**
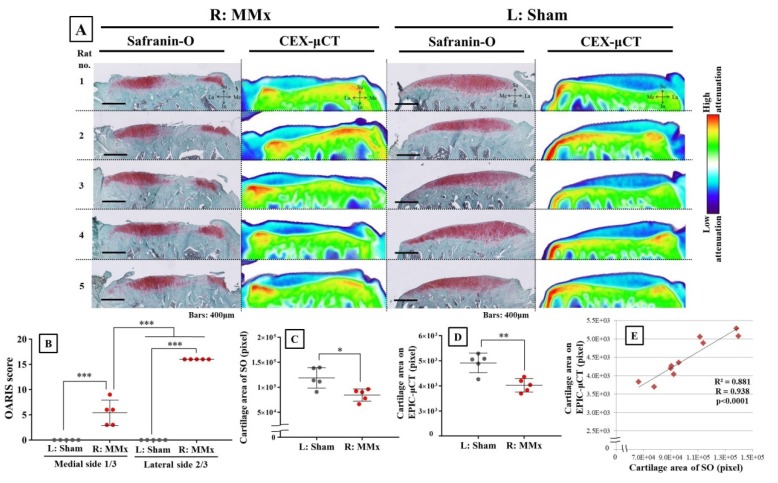
Confirmed degradation of articular cartilage by both Safranin-O (SO) staining and CEX-μCT imaging for all samples. (**A**) Comparison between Safranin-O staining and CEX-μCT imaging of articular cartilage on the tibia medial plateau. Both methods show cartilage degradation with similar morphology. (**B**) Osteoarthritis Research Society International (OARSI) guideline scores for Safranin-O staining in both MMx-treated and sham-treated groups. (**C**) Cartilage area based on Safranin-O-positive staining for both MMx-treated and sham-treated groups. (**D**) Cartilage area (pixels) according to CEX-μCT method for both MMx-treated and sham-treated groups. (**E**) Correction analysis of the cartilage area between both Safranin-O staining and CEX-μCT imaging methods. * *p* < 0.05, ** *p* < 0.01, *** *p* < 0.001.

Tissue staining of articular cartilage clearly demonstrated degeneration based on the decreased Safranin-O-positive area (Figure 5A). Moreover, CEX-μCT also revealed a reduced area of cartilage with matched CEX-μCT images and stained tissue images being consistent. In addition, the quantitative values of the cartilage area were significantly decreased on both tissue stain and CEX-μCT analyses (Figure 5C,D), which further supported the similarity between methods. The OARSI guideline scores were significantly increased in MMx-treated knees compared with those of sham-treated knees, which was more severe on the lateral two-thirds side of the MMx-treated knees (Figure 5B). Correlation analysis between cartilage areas obtained from Safranin-O staining and CEX-μCT imaging showed a strong significant correlation (*R* = 0.938) (Figure 5E).

### 3.5. MMx-Induced KOA Increased Total Cartilage Volumes 

**Figure 6:**
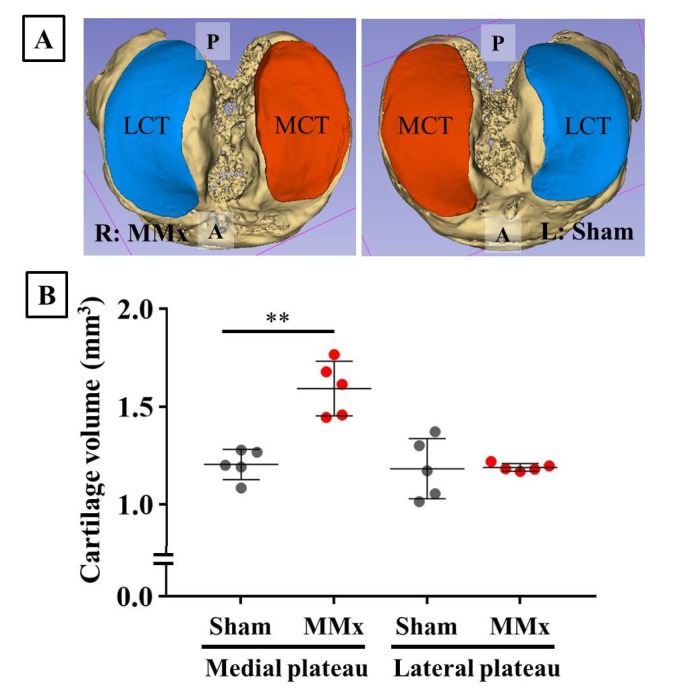
Reconstruction and volume calculation of the articular cartilage and subchondral bone using the CEX-μCT imaging method. (**A**) A representative case of reconstructed articular cartilage and subchondral bone. (**B**) Comparison of cartilage volumes between MMx-treated and sham-treated knees. The medial cartilage volumes were significantly increased in the MMx-treated knee compared with that in the sham-treated knee. Abbreviations: P, posterior; A, anterior; MCT, medial cartilage of the tibial plateau; LCT, lateral cartilage of the tibia plateau. ** *p* < 0.01 on paired *t*-test.

Using images from the CEX-μCT imaging method, the articular cartilage and subchondral bone were fully reconstructed as 3D images (Figure 6A). The cartilage volumes of the medial tibial plateau were significantly increased in the MMx-treated knees compared with those of the sham-treated knees (Figure 6B). In contrast, no changes were observed in the lateral cartilage volumes.

### 3.6. MMx-Treatment-Induced Gene Expression Associated with Knee Synovitis

**Figure 7:**
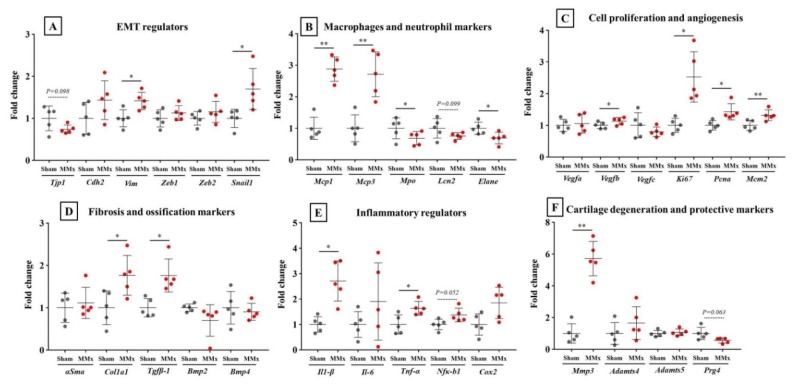
Synovitis-associated gene expression levels in the synovium. (**A**–**F**) Gene expression levels of synovitis-associated genes. * *p* < 0.05, ** *p* < 0.01 on paired *t*-test. EMT, epithelial–mesenchymal transition.

Analyses of the synovium using qPCR revealed that some gene expression associated with synovitis was significantly changed in the MMx-induced KOA knees compared with that in the sham-treated knees (Figure 7A–F). Among the differentially regulated genes, Mcp1 and Mcp3 are markers for macrophages (Figure 7B), Ki67 is a cell proliferation marker (Figure 7C), Cal1a1 and Tgfβ-1 are fibrillation markers (Figure 7D), IL-1β is an inflammatory cytokine (Figure 7E), and Mmp3 is a cartilage degradation maker (Figure 7F). All these genes reflected significant changes in expression levels, which reflected synovitis in the synovium of MMx-treated knees. Also, expression levels of Vim and Snail1, which are epithelial-mesenchymal transition markers, were significantly increased in the MMx-treated knees compared with those in the sham-treated knees (Figure 7A).

## 4. Discussion

In the current study, an aim was to establish the foundation for the CEX-μCT imaging method in order to use it to comprehensively evaluate articular cartilage. We determined that 30% Oypalomin was the optimal concentration to differentiate cartilage and bone. Subsequently, 3D images of the articular cartilage and subchondral bone obtained using the CEX-μCT method with 30% Oypalomin were constructed. The 3D images obtained with CEX-μCT showed the same morphology as that of the cartilage in the normal and MMx-induced KOA model based on comparing them to the more traditional macroscopic images. Also, the area of cartilage determined using the CEX-μCT method showed a strong correlation to the area measured using Safranin-O tissue staining. Moreover, the changes in cartilage localization in MMx-induced KOA could be detected using the CEX-μCT method. Therefore, the CEX-μCT method that was developed in this study reflected overall articular cartilage health with respect to evaluating cartilage. Furthermore, the CEX-μCT method with 30% Oypalomin may be comparable to the EPIC-μCT method reported by Xie et al. [11] and Willett et al. [12].

Previously, studies using KOA animal models have relied on tissue staining analyses that require relatively large amounts of time to obtain the microscopic images. For example, there are many processes that involve extensive time, such as paraffin embedding, tissue sectioning using a microtome or cryostat, and tissue staining. These not only require delicate finesse and professional techniques, but are also unforgiving, often making it difficult to be able to start over. Moreover, focusing on a thin slice method for paraffin-embedded and frozen blocks, it is difficult to exactly cut the same part between different samples, which may result in reproducibility problems. In contrast, when preparing cartilage samples for the CEX-μCT method developed in this study, there was no required technique that would hinder reproducibility. However, a crucial drawback to the CEX-μCT method is the requirement for a μCT apparatus, which is a specialized and expensive system.

It was the main aim of the current study to clarify the characteristics of degradations in articular cartilage, subchondral bone, and synovium in the knee complex of a post-traumatic OA animal model, in which KOA was induced by MMx, using the newly developed CEX-μCT method in this study. In the results, it was demonstrated that the cartilage degeneration and the inflammatory reactions occurring in the synovium were increased in the MMx-induced KOA model. On the other hand, no morphological degeneration of the subchondral bone was observed. Therefore, four weeks after the induction of the MMx-KOA model, it is suggested that no significant morphological changes are observed in the subchondral bone, but significant changes are observed in the articular cartilage and synovium.

It is well known that chronic inflammation activates macrophages, rather than neutrophils, and also affects fibrosis [16,17]. In the current study, gene expression in the synovium of MMx-induced KOA demonstrated increased expression levels for inflammatory cytokines Il-1β and Tnf-α, macrophage markers Mcp1 and Mcp3, and fibrosis markers Col1a1 and Tgfβ-1, without increased expression of neutrophil markers. Moreover, epithelial–mesenchymal transition (EMT) regulators Vim and Snail1 and cell growth markers Ki67, segmentation, and Mcm2 were also increased in MMx-induced KOA. EMT reactions leading to cell growth and the development of tumors are known to be exacerbated by chorionic inflammations [18,19]. Therefore, it is suggested that MMx-induced KOA may induce chronic inflammation.

Mmp3 expression was markedly increased in the synovium of MMx-induced KOA with 5.7-fold mean changes compared with that of the sham-treated knees. The matrix metalloproteinase-3 (MMP3) secretions from the synovium into the joint space are induced by NF-κB signaling in response to inflammatory reactions and can degrade articular cartilage [20]. Moreover, previous research has reported that increased MMP secretions from the synovium into the joint space in human KOA are associated with the pathogenesis of cartilage degradation [20,21,22,23]. Therefore, it can be hypothesized that, following chronic synovial inflammation, MMP3 secreted from the synovium may induce degradation of the cartilage in the MMx-induced KOA model, similar to that in human KOA. To confirm this hypothesis, experiments using the intra-articular administration of an MMP3 inhibitor would be necessary.

IL-1β expression was also increased in the synovium of MMx-induced KOA with a mean 2.7-fold change. IL-1β secreted from the synovium into the joint space plays a crucial role in the pathogenesis of KOA [24]. IL-1β induces NF-κB signaling and the expressions of catabolic proteins for cartilage, such as MMP3 and ADAMTS proteases, which further induces the expression of inflammatory cytokines, including IL-1β [25]. Therefore, it is possible that there is a signal axis such as “IL-1β-NF-κB-MMPs and IL-1β” in the MMx-induced KOA model that leads to the degradation of cartilage and an increase in synovitis.

The CEX-μCT method clarified some characteristics of the MMx-induced KOA model. In the local analysis, the comparison of the calculated area of cartilage between Safranin-O staining and CEX-μCT imaging demonstrated a strong correlation, which suggests robustness and accuracy for CEX-μCT in measuring cartilage area and volume. Therefore, we suggest that the CEX-μCT method may be useful for detecting the local area and total volume of cartilage. Furthermore, local analysis of the tibia plateau in MMx-induced KOA using CEX-μCT revealed significant decreases in the area of medial cartilage, even though the overall volumes were significantly increased. On the basis of these phenomena, it is possible that chondrocytes of the articular cartilage become hypertrophic in non-abrasion areas. Chondrocytes becoming hypertrophic is known to be a pathogenic factor of KOA [26]. Therefore, to precisely evaluate overall articular cartilage degradation, it is important to employ the combination of analysis on 3D images and local tomographic images using the CEX-μCT method. If only local evaluation using tissue staining is performed in drug discovery studies using KOA models, there is a possibility that actual medicinal effects are overlooked.

## 5. Conclusions

In summary, 30% Oypalomin as a contrast reagent was optimal for CEX-μCT imaging in this study. In addition, the characteristics of cartilage degradation in MMx-induced KOA were detected using CEX-μCT combined with classical methods. Finally, the synovium in MMx-induced KOA exhibited chronic inflammation, suggesting that it may contribute to cartilage degradation (the figure as graphical abstract). On the basis of the findings from the current study, we suggest that CEX-μCT will prove helpful in selecting optimal models and in discovering and testing drugs in future KOA studies.

## Supplementary Materials

Supplementary materials can be found at https://www.mdpi.com/2227-9059/8/3/56/s1.

## Abbreviations

CEX-μCT	contrast enhanced X-ray micro computed tomography
PBS	phosphate-balanced saline
P	posterior
A	anterior
MCT	medial cartilage of tibial plateau
LCT	lateral cartilage of tibial plateau
MMx	medial meniscectomy
KOA	knee osteoarthritis
qPCR	real-time quantitative polymerase chain reaction
